# Efficacy of lure mixtures in baited traps to attract different fruit fly species in guava and vegetable fields

**DOI:** 10.3389/finsc.2022.984348

**Published:** 2023-01-30

**Authors:** Shakil Ahmad, Coline C. Jaworski, Farman Ullah, Momana Jamil, Hayat Badshah, Farman Ullah, Yanping Luo

**Affiliations:** ^1^ School of Plant Protection, Hainan University, Haikou, Hainan, China; ^2^ Department of Plant Protection, the University of Agriculture, Peshawar, Pakistan; ^3^ Université Côte d’Azur, INRAE, CNRS, UMR ISA, Nice, France; ^4^ Beijing Academy of Agriculture and Forestry, Institute of Plant and Environment Protection, Beijing, China; ^5^ Department of Plant Biosecurity, College of Plant Protection, China Agricultural University, Beijing, China

**Keywords:** Tephritidae, cue lure, GF-120, methyl eugenol, 4-allyl-1, 2-dimethoxybenzene-carboxylate, 4-(p-acetoxyphenyl)-2-butanone

## Abstract

Fruit flies (Diptera: Tephritidae) are major pests of fruits and vegetables worldwide. We measured the efficacy of attractive lure mixtures in baited traps on naturally-occurring fruit flies in commercial mosaic guava and vegetables fields in Pakistan. We tested three mixtures (methyl-eugenol [ME] and cue lure [CL]; GF-120 and methyl eugenol; and GF-120 and cue lure) in eleven ratios: 0:100, 10:90, 20:80, 30:70, 40:60, 50:50, 60:40, 70:30, 80:20, 90:10, and 100:0. We recorded three fruit fly species: *Bactrocera zonata* was the most abundant in baited traps, followed by *Bactrocera dorsalis*, while *Zeugodacus cucurbitae* was significantly less attracted to baited traps. We also found that the most attractive mixture and ratio varied among species: *B. dorsalis* was most attracted by 40CL:60ME, while *B. zonata* was most and equally attracted by 100ME, 10CL:90ME, 20CL:80ME, 30CL:70ME, and 40CL:60ME. Finally, *Z. cucurbitae* was most attracted by 10CL:90ME, which resulted in the highest total number of flies counted in 10CL:90ME-baited traps. Mixtures with GF-120 were less attractive to all three species. Our results suggest that lure mixtures in baited traps influence the attraction of fruit flies in a species-specific way. This needs to be considered in the integrated pest management of multiple species of fruit flies simultaneously. If *Bactrocera* species are most damaging and abundant, a 40CL:60ME mixture in baited traps will likely be most effective to reduce pest abundance and crop damage. However, if *Z. cucurbitae* is the main pest target causing most crop damage and yield loss, 10CL:90ME-baited traps will be a more effective in their monitoring and management.

## Introduction

Fruit flies (Diptera: Tephritidae) are amongst most important pests of commercial fruits and vegetables worldwide (1–3). Fruit flies are polyphagous pest species attacking almost 40 different fruit and vegetable species. Fruit flies severely affect the commercial value of fruit and vegetables and cause up to 30-100% loss (4). They cause direct fruit damage, fruit dropping, and export market losses through quarantine restrictions (5). Currently, Pakistan is losing an estimated 200 million US dollars each year due to a lack of the latest technologies and failure to attain potential benefits from the existing protection measures (6). Fruit flies are responsible for 80% of losses in guava fruit crops (7). *Bactrocera zonata* is among the most devastating pest species causing 30-100% losses in fruit crops in Pakistan, and is found in all agricultural areas. Various tactics have been implemented to control fruit fly pests, but chemical applications remain the primary tool to manage fruit fly pests (8). However, broad-spectrum chemical applications lead to the development of insecticide resistance in insect pests (9) and are detrimental to non-target and beneficial insects (10).

Kairomones – volatile plant chemicals used by fruit flies to locate their host (11) – have been successfully exploited to monitor and manage fruit fly populations. The bait application technique (BAT) is an essential component of fruit fly management programs worldwide (12, 13). Various attractants like methyl eugenol (4-allyl-1,2-dimethoxybenzene-carboxylate) and another kairomone commercially known as cue lure (4-(p-acetoxyphenyl)-2-butanone) in male annihilation techniques (MAT) kill adult male flies before they mate with females, and therefore control the entire population in the target area for many years (14, 15). To control the female population, attractants like protein hydrolysate, orange ammonia, and liquid protein bait have also been tested (16). MAT is an effective management strategy for several *Bactrocera* species (17, 18). It was used to eradicate the oriental fruit fly *B. dorsalis* (Hendel) from Rota Island by distributing thousands of fiberboard blocks soaked with ME and an insecticide (19). Cue lure is also used in baited traps against *B. dorsalis* and the melon fly, *Z. cucurbitae* (20). Methyl eugenol combined with cue lure attracted twice as many males compared with traps baited with cue lure alone (21). The olfactory and phago-stimulatory effect of methyl eugenol is attractive to fruit flies from up to 800 m (22, 23). Methyl eugenol and cue lure baited traps were also used for a year-long monitoring to detect the initial infestation of *B. dorsalis* and *Z. cucurbitae* in a macadamia nut orchard in Southern California (24). Cue lure alone with an insecticide has been used to control populations of *Z. cucurbitae* in Mikayo Island; however it is less attractive and therefore less effective in MAT programs (25). A protein bait (Nu-Lure; Miller Chemical and Fertilizer Co., Hanover, PA) has been used as a standard bait in Mexico and California for fruit fly detection and control (26). Control programs for *Bactrocera* species are based on male lures to detect and suppress pest populations, despite the potential side effects of lures on beneficial arthropods (27). Therefore, it is important to use the most efficient mixture in baits with lowest effects on non-target species. This is especially important where such methods could be used to control several fruit fly pest species simultaneously.

Guava (*Psidium guajava* L. Family Myrtaceae) is a perennial, highly palatable, and nutritious tropical and sub-tropical fruit adapted to a wide range of climatic and soil conditions (28). It is believed that guava originated in Mexico or Central America and has a well-established market in about 60 countries due to its rusticity and productivity (29). Currently, the major guava producer countries are South Asia, the Islands of Hawaii, Cuba, Brazil, Pakistan, and India (29). Guava is the fourth most important fruit in Pakistan and was grown over an area of 62,500 ha with a production of 555,300 tons in the year 2006-2007 (30). It is mainly grown in Shariqpur, Kasur, Lahore, Sheikhupora, Shangla, Kohat, Haripur, Bannu, Gujranwala, Larkana, and Hyderabad. In Pakistan, various commercial cultivars including Gola, Surakhi, Chota Gola, Choti Surakhi, Ramzani, Karela, Baidana, Surkha, Lal Badshah, Sdabahar, Hafsi, Sufaida are available in the market (31, 32). Another important vegetable produced in Pakistan is luffa (*Luffa cylindrica* L.). It is a creeping plant known as sponge gourd, predominantly found in tropical and subtropical areas (33). During 2008-2009, the total area grown with luffa was 20,982 hectares, with a total annual production of 20,982 tons in Pakistan (34). Although there are large numbers of luffa cultivars grown, there is a large production gap between Pakistan and international yields due to outdated cultural techniques, increasing weed infestations, gregarious pest attacks and increasing insect resistance to insecticides, among other factors (35). During the vegetative growth and production phases, several insect pests attack luffa, including squash bugs, squash vine borers, cucumber beetles, red pumpkin beetle, ants, thrips, and fruit flies (36). In District Kohat (Pakistan), guava is grown on large acreage. However in summer, cucurbitaceous vegetables, and mainly luffa, are grown for local use and surrounding markets.

Since guava and summer vegetables such as luffa are important summer food sources in Pakistan, efficient pest control programs such as MAT targeting fruit flies in multiple crops are essential. In the present study, we investigated the attractiveness of different mixtures of methyl eugenol and cue lure to fruit flies, and the efficacy of traps baited with these mixtures and the commercial bait GF-120 to control fruit flies.

## Materials and methods

The field experiment was conducted in two joint commercial fields of ~0.2 ha each in Kohat City, Khyber Pakhtunkhwa, Pakistan (33” 37’ 20” N latitude, 71” 55’ 20” E longitude). Each field was a fine-scale mosaic of crops with guava and luffa as the main crops (> 95% of the total area; ~48% guava and ~48% luffa), while the rest was a diversity of vegetables. We tested three kairomone mixtures: (1) methyl eugenol + cue lure; (2) GF-120 + methyl eugenol; and (3) GF-120 + cue lure. GF-120 is a commercial bait composed of the insecticide spinosad, a microbial hydrolyzed protein, sugars, adjuvants and a series of conditioners; it is formulated to have both an attractant and feeding stimulant function (37). Each mixture was tested in eleven ratios: 100:0, 90:10, 80:20, 70:30, 60:40, 50:50, 40:60; 30:70, 20:80, 10:90, and 0:100. Mixtures were completed with sugar (10% of final mass) and insecticide Dipterex (5% of final mass). Three replicates of each ratio and each mixture were prepared. Baited traps consisted of white round plastic bowls (volume 1.4 L) attached with iron wire to poles at height 1.5-2 m above ground; they were placed in the shade 30 **m** apart in fields. A cotton wick impregnated with the kairomone mixture was placed inside the trap using a wire hook. Mixtures and ratios were randomly assigned to traps. The experiment was conducted near the time of fruit ripening and lasted six weeks. Every week, the number of flies of each species (*B. dorsalis, B. zonata*, or *Z. cucurbitae*) in each trap was counted, and the lure was replaced with a fresh one.

All statistical analyses were performed using R Core Team (38). The effect of baits on flies caught in baited traps weekly was assessed for each species separately, using linear mixed models (function ‘lmer’; R library ‘lme4’; 39) with the trap ID as random effect to account for repeated measures through time. Because of the high number of different bait treatments (11 ratios × 3 mixtures = 33 treatments), the test for each species was run on a subset of data from the most attractive baits, that is baits that attracted in average more flies than the average across all baits (*B. dorsalis*: seven treatments; *B. zonata*: 10 treatments; *Z. cucurbitae*: 12 treatments). The treatment (mixture and ratio) was used as fixed effect, and the significance of the treatment effect was tested with an ANOVA with a χ^2^-test. The normality and homoscedasticity of residuals was verified using the ‘plot(simulateResiduals())’ function (R library ‘DHARMa’; 40). Mean comparisons between treatments were performed using the ‘emmeans’ function (R library ‘emmeans’; 41). The crop type (guava or luffa) was not used as fixed effect because the fields were a mosaic of small patches of both crops. The field was not used as random effect because the two experiment fields were adjacent and we considered it as one big field. Week-to-week variation was extremely small (mean number of flies of each species caught per trap weekly: 2.22; mean weekly SE across each bait treatment and species: 0.42). Finally, we tested whether the number of flies caught weekly differed between species independently of the bait treatment: we performed a generalized linear model with a negative binomial error distribution (function (glmer.nb’; R library ‘lme4’) to account for data overdispersion, using the species as fixed effect (either *B. dorsalis, B. zonata* or *Z. cucurbitae*), and the trap ID as random effect.

## Results

**Figure 1:**
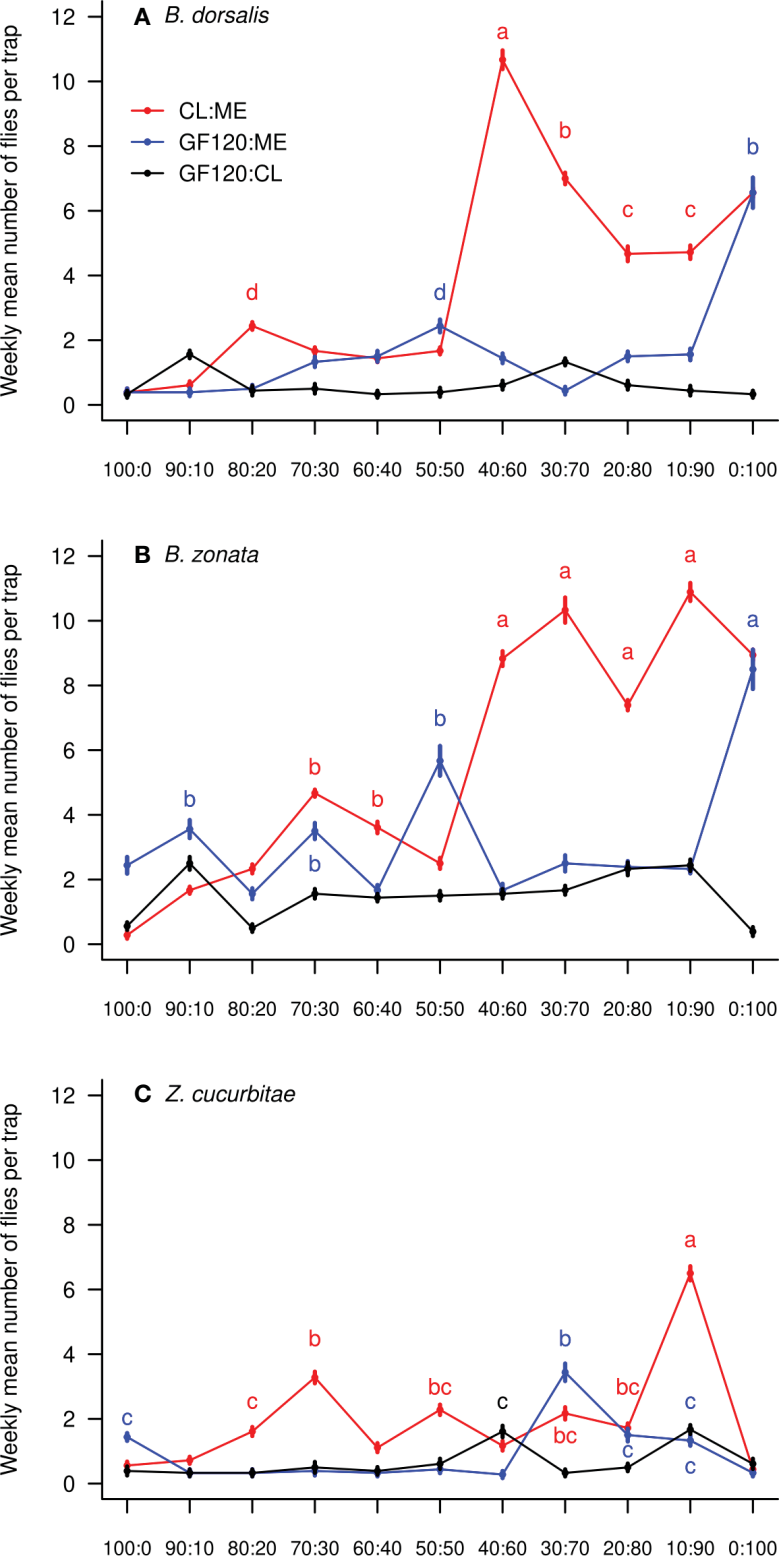
Weekly mean (± SE) number of fruit flies collected in traps baited with the three different mixtures and eleven different ratios: **(A)** *B. dorsalis*; **(B)** *B. zonata*; **(C)** *Z. cucurbitae*. In each panel, different letters show significant differences between ratios from the same mixture and between different mixtures (**Tables S1-3**); letters are colored following their corresponding mixture. Data points without letters were not included in the comparisons. ‘ME’: methyl eugenol; ‘CL’: cue lure (4-(p-acetoxyphenyl)-2-butanone); ‘GF’: GF120 (insecticide).

**Figure 2:**
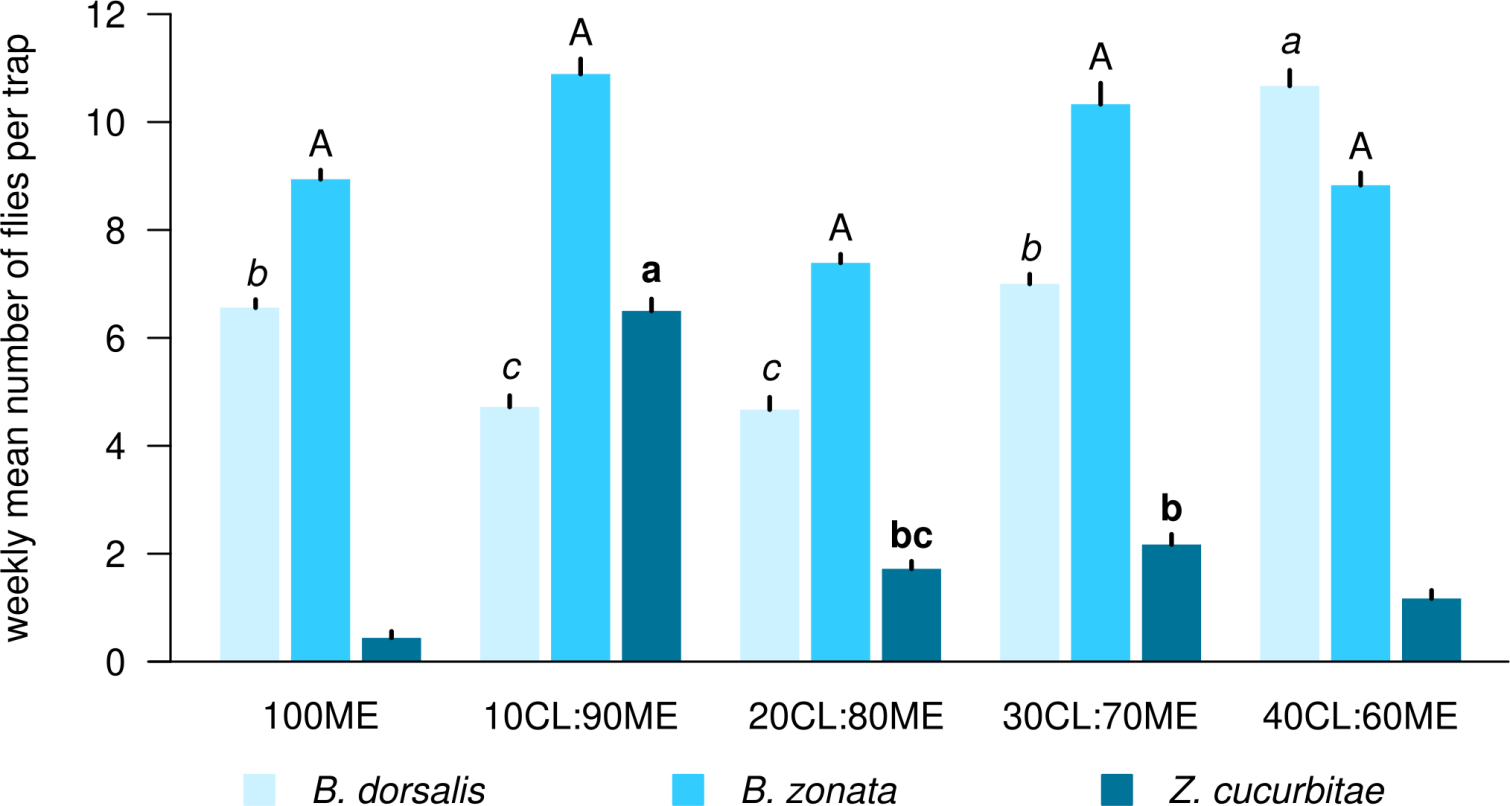
Comparison of mean number of flies caught weekly between species among the most efficient mixtures and ratios for each species. Different letters with the same font show significant differences between treatments for each species (reported from **Figure 1**). ‘ME’: methyl eugenol; ‘CL’: cue lure (4-allyl-1,2-dimethoxybenzene-carboxylate); ‘GF’: GF120 (insecticide).

There was a significant effect of the treatment (mixture and ratio) on the number of flies caught weekly per trap in all three species (**Table 1**). **Figure 1** shows the weekly mean (± SE) number of flies of each species per trap in each bait treatment, while **Figure 2** compares mean (± SE) numbers of flies caught per trap weekly across species, for the most attractive treatments of each species. The number of *B. dorsalis* flies caught weekly was highest in 40CL:60ME-baited traps (10.7 ± 0.2 flies caught weekly), and the second most attractive baits to *B. dorsalis* were 30CL:70ME (7.0 ± 0.2) and 100ME (6.6 ± 0.2). The number of *B. zonata* flies caught weekly was found to be highest and equally high in the bait treatments 100ME (8.9 ± 0.2), 10CL:90ME (10.9 ± 0.3), 20CL:80ME (7.4 ± 0.1), 30CL:70ME (10.3 ± 0.2), and 40CL:60ME (8.8 ± 0.2). Finally, the number of *Z. cucurbitae* flies was highest in 10CL:90ME baited traps (6.5 ± 0.2), and the second most attractive baits were 30CL:70ME (2.2 ± 0.2) and 20CL:80ME (1.7 ± 0.1). The number of flies caught weekly in each trap was significantly different across species independently of the treatment (χ^2^ = 315, df = 2, P < 0.001): numbers of *B. zonata* were highest (mean: 3.4 ± 0.1), followed by *B. dorsalis* (2.0 ± 0.1) and the number of *Z. curcubitae* was the lowest (1.1 ± 0.1).

**Table 1:** Effects of the bait treatment (mixture and ratio) on the number of flies caught weekly per trap in each species.

Effect of the bait treatment	χ^2^	d.f.	*P*
*B. dorsalis*	68.6	6	< 0.001 ***
*B. zonata*	67.5	9	< 0.001 ***
*Z. cucurbitae*	85.4	11	< 0.001 ***

*** indicates statistical significance P < 0.001.

## Discussion

We tested the attractiveness of three mixtures (methyl eugenol with cue lure, GF120 with methyl eugenol and GF120 with cue lure) in eleven different ratios (from 0:100 to 100:0) to fruit fly pests in commercial farms in Kohat, Pakistan. We detected three species: *B. dorsalis*, *B. zonata* and *Z. cucurbitae*, known to damage numerous fruit and vegetable crops in the area (42, 43). While *B. zonata* was caught in highest numbers in baited traps followed by *B. dorsalis* and *Z. cucurbitae*, we found that the most attractive mixture to all three species was methyl eugenol (ME) with cue lure (CL), although this varied in ratio. While *B. zonata* was equally attracted by 100ME, 10CL:90ME, 20CL:80ME, 30CL:70ME and 40CL:60ME, *B. dorsalis* was most attracted by 40CL:60ME. Finally, *Z. cucurbitae* was most attracted by 10CL:90ME, although in lower numbers than *B. dorsalis*.

Our results are consistent with the hypothesis that mixing two lures in different ratios may enhance the bait attractiveness (44, 45). Differences in attraction to specific ratios of mixtures between species were also reported previously. Previous studies – which did not test the attractiveness of mixtures in the same ratios – reported that *B. dorsalis* was mostly attracted by pure methyl eugenol (20, 46). We also found a high attractiveness of pure methyl eugenol to *B. dorsalis*, but we show that the 40CL:60ME mixture was even more attractive. This mixture could be a fine tune to a preferred host plant kairomone signal in the area. Similar to our results, *Z. cucurbitae* was previously described as mostly unresponsive to different ratios of methyl eugenol and cue lure (20, 46), except pure methyl eugenol which was unattractive (47).

We found only three fruit flies species caught in baited traps, with *B. zonata* the most abundant one, closely followed by *B. dorsalis*, while *Z. cucurbitae* was less abundant in traps. These results are similar to the findings of Ullah et al. (44) in guava orchards from Kohat. This does not necessarily mean that *Z. cucurbitae* was not abundant in the experiment’s area, but that it was less attracted by the mixtures provided in baited traps. 44 also reported a negligible number of *Z. cucurbitae* flies in GF-120 baited traps set in melons orchards in Badghis, Afghanistan. Our results suggest that a mixture of GF-120 and cue lure may be more attractive to *Z. cucurbitae* rather than GF-120 used alone. Consistent to our findings, pure cue lure was previously reported to be less attractive to *Z. cucurbitae* than a mixture of cue lure with methyl eugenol (48). Traps baited with mixtures of methyl eugenol with cue lure were also previously reported to be more efficient at trapping *Bactrocera tryoni* compared to traps baited with pure lures (49).

We show that there isn’t a single mixture that is most attractive to all three fruit fly species among those recorded in our study. 40CL:60ME is the most efficient mixture to attract both *B. dorsalis* and *B. zonata*, but 10CL:90ME is the most attractive mixture to *Z. cucurbitae* and therefore led to the highest total number of flies caught in traps. Therefore, the optimization of the bait mixture in local fruit fly pest control programs will depend on the relative abundance of these three fly species, and their relative economic impacts. If *Bactrocera* species cause most damage in the target crop, we recommend using 40CL:60ME as bait mixture. If, however, *Z. cucurbitae* is the main pest target, we recommend using 10CL:90ME.

Future research should further fine-tune the cue lure – methyl eugenol mixture that best matches target crop kairomone emissions and that is most attractive to all species, since insects are likely to respond to very specific kairomone ratios. To that end, it would be useful to measure the plant volatile emissions of the crops targets of pest control programs. The most attractive mixture ratios found here could then be used as starting points to fine tune mixtures. Testing tri-lure mixtures could also be an effective option in a multi-pest species system. This will require measuring differences between relative attractiveness (number of flies of each specie caught in baited traps) and relative local abundances, which should be measured independently based for instance on passive trapping or plant-based counts. Finally to measure the efficacy of baited traps as a pest control method, it will be important to measure the relative abundance of fruit flies as well as crop damage and yields *in situ* in the presence or absence of baited trap.

## Data availability statement

The original contributions presented in the study are included in the article/**Supplementary Material**. Further inquiries can be directed to the corresponding author.

## Author contributions

SA, FU (3rd author), HB and YL: conceptualization, resources, project administration, and funding acquisition. SA, CCJ, FU (6th author) and MJ: Formal analysis and writing original draft preparation. All authors contributed to the article and approved the submitted version.
