# Supplementary material for: Efficacy of lure mixtures in baited traps to attract different fruit fly species in guava and vegetable fields
Source: Front Insect Sci. 2023 Jan 30;2:984348. doi: 10.3389/finsc.2022.984348 (PMC10926387; doi:10.3389/finsc.2022.984348)
Supplement: Supplementary file 1 [file Table_1.docx]

**Supplementary material for the article:**

Efficacy of mixtures of parapheromone lures on trapping different fruit fly species in guava orchards and summer vegetables

BY: Shakil Ahmad, Coline C. Jaworski, Farman Ullah, Momana Jamil, Hayat Badshah, Farman Ullah and Yanping Luo

**Table S1.** Mean comparisons of the number of *B. dorsalis* flies caught weekly per trap between bait treatments (mixture and ratio) by the function ‘emmeans’ (R library ‘emmeans’; Lenth, 2022).

| Contrast | Estimate | SE | df | t.ratio | *P* |
| --- | --- | --- | --- | --- | --- |
| 100ME – 10CL:90ME  100ME – 20CL:80ME  100ME – 30CL:70ME  100ME – 40CL:60ME  100ME – 80CL:20ME  100ME – 50GF:50ME  10CL:90ME – 20CL-80ME  10CL:90ME – 30CL:70ME  10CL:90ME – 40CL:60ME  10CL:90ME – 80CL:20ME  10CL:90ME – 50GF:50ME  20CL:80ME – 30CL:70ME  20CL:80ME – 40CL:60ME  20CL:80ME – 80CL:20ME  20CL:80ME – 50GF:50ME  30CL:70ME – 40CL:60ME  30CL:70ME – 80CL:20ME  30CL:70ME – 50GF:50ME  40CL:60ME – 80CL:20ME  40CL:60ME – 50GF:50ME  80CL:20ME – 50GF:50ME | 1.83  1.89  -0.444  -4.11  4.11  4.11  0.0556  -2.28  -5.94  2.28  2.28  -2.33  -6.00  2.22  2.22  -3.67  4.56  4.56  8.22  8.22  0 | 0.52  0.52  0.52  0.52  0.52  0.52  0.604  0.60  0.60  0.60  0.60  0.60  0.60  0.60  0.60  0.60  0.60  0.60  0.60  0.60  0.60 | 17  17  17  17  17  17  17  17  17  17  17  17  17  17  17  17  17  17  17  17  17 | 3.50  3.61  -0.849  -7.85  7.85  7.85  0.0920  -3.77  -9.84  3.77  3.77  -3.86  -9.93  3.68  3.68  -6.07  7.54  7.54  13.6  13.6  0 | 0.035 *  0.029 *  0.98  < 0.001 ***  < 0.001 ***  < 0.001 ***  1  0.021 *  < 0.001 ***  0.021 *  0.021 *  0.017 *  < 0.001 ***  0.025 *  0.025 *  < 0.001 ***  < 0.001 ***  < 0.001 ***  < 0.001 ***  < 0.001 ***  1 |

* *P* < 0.05; *** *P* <0.001.

**Table S2.** Mean comparisons of the number of *B. zonata* flies caught weekly per trap between bait treatments (mixture and ratio) by the function ‘emmeans’ (R library ‘emmeans’; Lenth, 2022).

| Contrast | Estimate | SE | df | t.ratio | *P* |
| --- | --- | --- | --- | --- | --- |
| 100ME – 10CL:90ME  100ME – 20CL:80ME  100ME – 30CL:70ME  100ME – 40CL:60ME  100ME – 60CL:40ME  100ME – 70CL:30ME  100ME – 50GF:50ME  100ME – 70GF:30ME  100ME – 90GF:10ME  10CL:90ME – 20CL:80ME  10CL:90ME – 30CL:70ME  10CL:90ME – 40CL:60ME  10CL:90ME – 60CL:40ME  10CL:90ME – 70CL:30ME  10CL:90ME – 50GF:50ME  10CL:90ME – 70GF:30ME  10CL:90ME – 90GF:10ME  20CL:80ME – 30CL:70ME  20CL:80ME – 40CL:60ME  20CL:80ME – 60CL:40ME  20CL:80ME – 70CL:30ME  20CL:80ME – 50GF:50ME  20CL:80ME – 70GF:30ME  20CL:80ME – 90GF:10ME  30CL:70ME – 40CL:60ME  30CL:70ME – 60CL:40ME  30CL:70ME – 70CL:30ME  30CL:70ME – 50GF:50ME  30CL:70ME – 70GF:30ME  30CL:70ME – 90GF:10ME  40CL:60ME – 60CL:40ME  40CL:60ME – 70CL:30ME  40CL:60ME – 50GF:50ME  40CL:60ME – 70GF:30ME  40CL:60ME – 90GF:10ME  60CL:40ME – 70CL:30ME  60CL:40ME – 50GF:50ME  60CL:40ME – 70GF:30ME  60CL:40ME – 90GF:10ME  70CL:30ME – 50GF:50ME  70CL:30ME – 70GF:30ME  70CL:30ME – 90GF:10ME  50GF:50ME – 70GF:30ME  50GF:50ME – 90GF:10ME  70GF:30ME – 90GF:10ME | -2.17  1.33  -1.61  -0.111  5.11  4.06  3.06  5.22  5.17  3.50  0.556  2.06  7.28  6.22  5.22  7.39  7.33  -2.94  -1.44  3.78  2.72  1.72  3.89  3.83  1.50  6.72  5.67  4.67  6.83  6.78  5.22  4.17  3.17  5.33  5.28  -1.06  -2.06  0.111  0.0556  -1.00  1.17  1.11  2.17  2.11  -0.0556 | 0.87  0.87  0.87  0.87  0.87  0.87  0.87  0.87  0.87  1.01  1.01  1.01  1.01  1.01  1.01  1.01  1.01  1.01  1.01  1.01  1.01  1.01  1.01  1.01  1.01  1.01  1.01  1.01  1.01  1.01  1.01  1.01  1.01  1.01  1.01  1.01  1.01  1.01  1.01  1.01  1.01  1.01  1.01  1.01  1.01 | 23  23  23  23  23  23  23  23  23  23  23  23  23  23  23  23  23  23  23  23  23  23  23  23  23  23  23  23  23  23  23  23  23  23  23  23  23  23  23  23  23  23  23  23  23 | -2.49  1.53  -1.85  -0.128  5.87  4.66  3.51  6.00  5.93  3.48  0.552  2.04  7.24  6.19  5.19  7.35  7.29  -2.93  -1.44  3.76  2.71  1.71  3.87  3.81  1.49  6.69  5.64  4.64  6.80  6.74  5.19  4.14  3.15  5.30  5.25  -1.050  -2.044  0.110  0.0550  -0.994  1.16  1.11  2.16  2.10  -0.0550 | 0.32  0.87  0.70  1  < 0.001 ***  0.0035 **  0.048 *  < 0.001 ***  < 0.001 ***  0.051  1  0.58  < 0.001 ***  < 0.001 ***  0.0010 ***  < 0.001 ***  < 0.001 ***  0.15  0.90  0.028 *  0.23  0.78  0.022 *  0.025 *  0.88  < 0.001 ***  < 0.001 ***  0.0037 **  < 0.001 ***  < 0.001 ***  0.0010 ***  0.012 *  0.10  < 0.001 ***  < 0.001 ***  0.99  0.58  1  1  0.99  0.97  0.98  0.51  0.55  1 |

* *P* < 0.05; ** *P* <0.01; *** *P* <0.001.

**Table S3.** Mean comparisons of the number of *Z. cucurbitae* flies caught weekly per trap between bait treatments (mixture and ratio) by the function ‘emmeans’ (R library ‘emmeans’; Lenth, 2022).

| Contrast | Estimate | SE | df | t.ratio | *P* |
| --- | --- | --- | --- | --- | --- |
| 10CL:90ME – 20CL:80ME  10CL:90ME – 30CL:70ME  10CL:90ME – 50CL:50ME  10CL:90ME – 70CL:30ME  10CL:90ME – 80CL:20ME  10CL:90ME – 10GF:90CL  10CL:90ME – 40GF:60CL  10CL:90ME – 10GF:90ME  10CL:90ME – 100GF  10CL:90ME – 20GF:80ME  10CL:90ME – 30GF:70ME  20CL:80ME – 30CL:70ME  20CL:80ME – 70CL:30ME  20CL:80ME – 50CL:50ME  20CL:80ME – 80CL:20ME  20CL:80ME – 10GF:90CL  20CL:80ME – 40GF:60CL  20CL:80ME – 10GF:90ME  20CL:80ME – 100GF  20CL:80ME – 20GF:80ME  20CL:80ME – 30GF:70ME  30CL:70ME – 50CL:50ME  30CL:70ME – 70CL:30ME  30CL:70ME – 80CL:20ME  30CL:70ME – 10GF:90CL  30CL:70ME – 40GF:60CL  30CL:70ME – 10GF:90ME  30CL:70ME – 100GF  30CL:70ME – 20GF:80ME  30CL:70ME – 30GF:70ME  50CL:50ME – 70CL:30ME  50CL:50ME – 80CL:20ME  50CL:50ME – 10GF:90CL  50CL:50ME – 40GF:60CL  50CL:50ME – 10GF:90ME  50CL:50ME – 100GF  50CL:50ME – 20GF:80ME  50CL:50ME – 30GF:70ME  70CL:30ME – 80CL:20ME  70CL:30ME – 10GF:90CL  70CL:30ME – 40GF:60CL  70CL:30ME – 10GF:90ME  70CL:30ME – 100GF  70CL:30ME – 20GF:80ME  70CL:30ME – 30GF:70ME  80CL:20ME – 10GF:90CL  80CL:20ME – 40GF:60CL  80CL:20ME – 10GF:90ME  80CL:20ME – 100GF  80CL:20ME – 20GF:80ME  80CL:20ME – 30GF:70ME  10CL:90ME – 40GF:60CL  10CL:90ME – 10GF:90ME  10CL:90ME – 100GF  10CL:90ME – 20GF:80ME  10CL:90ME – 30GF:70ME  40CL:60ME – 10GF:90ME  40CL:60ME – 100GF  40CL:60ME – 20GF:80ME  40CL:60ME – 30GF:70ME  10CL:90ME – 100GF  10CL:90ME – 20GF:80ME  10CL:90ME – 30GF:70ME  100GF – 20GF:80ME  100GF – 30GF:70ME  20GF:80ME – 30GF:70ME | 4.78  4.33  4.22  4.22  4.89  4.83  4.89  5.17  5.06  5.00  3.06  -0.444  -0.556  -1.56  0.111  0.056  0.111  0.389  0.278  0.222  -1.72  -0.111  -1.11  0.556  0.500  0.556  0.833  0.722  0.667  -1.28  -1.00  0.667  0.611  0.667  0.944  0.833  0.778  -1.17  1.67  1.61  1.67  1.94  1.83  1.78  -0.167  -0.056  0  0.278  0.167  0.111  -1.83  0.056  0.333  0.222  0.167  -1.78  0.278  0.167  0.111  -1.83  -0.111  0.167  -2.11  -0.056  -2.00  -1.94 | 0.45  0.45  0.45  0.45  0.45  0.45  0.45  0.45  0.45  0.45  0.45  0.452  0.452  0.45  0.452  0.452  0.452  0.452  0.452  0.452  0.45  0.452  0.45  0.452  0.452  0.452  0.452  0.452  0.452  0.45  0.45  0.452  0.452  0.452  0.452  0.452  0.452  0.45  0.45  0.45  0.45  0.45  0.45  0.45  0.452  0.452  0  0.452  0.452  0.452  0.45  0.452  0.452  0.452  0.452  0.45  0.452  0.452  0.452  0.45  0.452  0.452  0.45  0.452  0.45  0.45 | 24  24  24  24  24  24  24  24  24  24  24  24  24  24  24  24  24  24  24  24  24  24  24  24  24  24  24  24  24  24  24  24  24  24  24  24  24  24  24  24  24  24  24  24  24  24  24  24  24  24  24  24  24  24  24  24  24  24  24  24  24  24  24  24  24  24 | 10.6  9.59  9.34  7.13  10.8  10.7  10.8  11.4  11.2  11.1  6.76  -0.983  -1.23  -3.44  0.246  0.123  0.246  0.861  0.615  0.492  -3.811  -0.246  -2.46  1.23  1.11  1.23  1.84  1.60  1.48  -2.83  -2.21  1.48  1.35  1.48  2.09  1.84  1.72  -2.58  3.69  3.57  3.69  4.30  4.06  3.93  -0.369  -0.123  0  0.615  0.369  0.246  -4.06  0.123  0.738  0.492  0.369  -3.93  0.615  0.369  0.246  -4.06  -0.246  -0.369  -4.67  -0.123  -4.43  -4.30 | < 0.001 ***  < 0.001 ***  < 0.001 ***  < 0.001 ***  < 0.001 ***  < 0.001 ***  < 0.001 ***  < 0.001 ***  < 0.001 ***  < 0.001 ***  < 0.001 ***  1  0.98  0.071  1  1  1  1  1  1  0.032 *  1  0.41  0.98  0.99  0.98  0.78  0.89  0.93  0.23  0.56  0.93  0.96  0.93  0.63  0.78  0.84  0.34  0.042 *  0.055  0.042 *  0.010 *  0.018 *  0.024 *  1  1  1  1  1  1  0.018 *  1  1  1  1  0.024 *  1  1  1  0.018 *  1  1  0.0043 **  1  0.0077 **  0.010 * |

* *P* < 0.05; ** *P* <0.01; *** *P* <0.001.

**REFERENCES**

Lenth R (2022). emmeans: Estimated Marginal Means, aka Least-Squares Means. R package version 1.8.1-1, <https://CRAN.R-project.org/package=emmeans>.
